# Effects of several flavonoids on human gut microbiota and its metabolism by *in vitro* simulated fermentation

**DOI:** 10.3389/fmicb.2023.1092729

**Published:** 2023-02-02

**Authors:** Lixia Pan, Hangyu Ye, Xionge Pi, Wei Liu, Zhao Wang, Yinjun Zhang, Jianyong Zheng

**Affiliations:** ^1^College of Biotechnology and Bioengineering, Zhejiang University of Technology, Hangzhou, China; ^2^Institute of Plant Protection and Microbiology, Zhejiang Academy of Agricultural Sciences, Hangzhou, China

**Keywords:** flavonoids, gut microbiota, gas, short-chain fatty acids, *in vitro* simulated fermentation

## Abstract

**Introduction:**

Flavonoids have antiviral, antitumor, anti-inflammatory, and other biological activities. They have high market value and are widely used in food and medicine fields. They also can regulate gut microbiota and promote human health. However, only a few flavonoids have been reported for their regulatory effects on human gut microbiota.

**Methods:**

The effects of hesperidin, hesperetin-7-O-glucoside, hesperetin, naringin, prunin, naringenin, rutin, isoquercitrin, and quercetin on gut microbiota structural and metabolic differences in healthy subjects were studied by means of *in vitro* simulated fermentation technology.

**Results:**

Results showed that the nine kinds of flavonoids mentioned above, especially hesperetin-7-O-glucoside, prunin, and isoquercitrin, were found to have more effect on the structure of human gut microbiota, and they could significantly enhance *Bifidobacterium* (*p* < 0.05). After 24 h of *in vitro* simulated fermentation, the relative abundance of intestinal probiotics (e.g., *Lactobacillus*) was increased by the three flavonoids and rutin. Furthermore, the relative abundance of potential pathogenic bacteria was decreased by the addition of hesperetin-7-O-glucoside, naringin, prunin, rutin, and isoquercitrin (e.g., *Lachnoclostridium* and *Bilophila*). Notably, prunin could also markedly decrease the content of H_2_S, NH_3_, and short-chain fatty acids. This performance fully demonstrated its broad-spectrum antibacterial activity.

**Discussion:**

This study demonstrates that flavonoids can regulate the imbalance of gut microbiota, and some differences in the regulatory effect are observed due to different structures. This work provides a theoretical basis for the wide application of flavonoids for food and medicine.

## 1. Introduction

In recent years, the important impact of gut microbiota on human health and disease has attracted widespread attention (Sarkar et al., 2021; Ling et al., 2022). Increasing evidence shows that changes in the gut microbiota not only cause various gastrointestinal diseases (Guinane and Cotter, 2013), but also are associated with other chronic diseases, such as metabolic syndrome (Lim et al., 2017), Parkinson’s disease (Castelli et al., 2021), and certain cancers (Li and Chen, 2022). The structure and function of the gut microbiota are influenced by endogenous and exogenous factors. Among the many influential factors, dietary regulation is considered a critical factor (Tremaroli and Bäckhed, 2012; Nie et al., 2018). The gut microbiota fully utilizes its metabolic capacity to catabolize and employ dietary factors, and the metabolites it produces can affect the host directly or indirectly (Trakman et al., 2022). The gut microbiota produces a variety of metabolites, including gases and short-chain fatty acids (SCFAs). Gases are primarily produced by anaerobic bacteria in the gut microbiota (Triantafyllou et al., 2014). Beneficial bacteria in the gut microbiota produce SCFAs, and SCFAs can mediate the interaction between the gut microbiota and the body (Serino, 2019). The type and amount of SCFAs and gases produced by the gut microbiota play an important role in maintaining its homeostasis (Kalantar-Zadeh et al., 2019). Flavonoids, which are widely distributed in plants, are dietary polyphenols with anti-oxidation and anti-inflammation biological activities (Kumar and Pandey, 2013). Most natural flavonoids are in the form of glycosides, which cannot be absorbed by the body as effectively as aglycones. Except for a small portion of the flavonoid glycosides in the daily diet, which are hydrolyzed into aglycones by enzymes in the digestive tract, most of them are transformed into aglycones by the gut microbiota in the colon and combined with the body’s own function (Sandoval et al., 2020). They are further metabolized into various metabolites and small-molecule phenolic acids that can be absorbed by the intestinal cells (Liu et al., 2018). Consequently, this biotransformation process of flavonoids mediated by gut microbiota can effectively improve the bioavailability of flavonoid glycosides.

Flavonoids are a class of compounds with 2-phenyl chromogenic ketones as the parent nucleus and can be divided into the subclasses of flavones, flavonols, flavanones, flavan-3-ols, anthocyanins, dihydroflavonols, isoflavones, and chalconoids (Santos-Buelga and Feliciano, 2017). Flavanones mainly include hesperetin, naringenin, erodcyol, and etc., meanwhile quercetin is one of the most common flavonols (Jiang et al., 2020). Hesperetin-7-O-glucoside, prunin, and isoquercitrin are flavonoid monoglucoside forms of hesperetin, naringenin, and quercetin, respectively. Hesperidin, naringin, and rutin are their corresponding flavonoid diglycosides. The structural formulae and related information of the nine flavonoids are shown in Table 1. Naringenin and quercetin have inhibitory effects on some common bacteria in the gastrointestinal tract, including *Lactobacillus rhamnosus* (probiotics), *Staphylococcus aureus* and *Salmonella typhimurium* (enteropathogens), and *Escherichia coli* (commensal bacteria; Parkar et al., 2008). Duda-Jodak assessed the impacts of various polyphenols on the gut microbiota representatives (*Lactobacillus*, *Bacteroides galacturonicus*, and *Ruminococcus gauvreauii*), and then concluded that concentration more than 250 μg/mL of hesperetin, naringenin, and 4–50 μg/mL of quercetin inhibited their growth (Duda-Chodak, 2012). Gwiazdowska et al. (2015) found that *Bifidobacterium adolescentis* and *Bifidobacterium bifidum* were sustained inhibited by hesperidin and quercetin. In addition to broad-spectrum antibacterial activity, flavonoids have the effect of increasing the proportion of beneficial bacteria in the intestinal tract. *In vivo* studies found that hesperidin increased the *Lactobacillus*/*Enterococcus* ratio, and decreased the *Clostridium coccoides*/*Eubacterium rectale* ratio in the Lewis rat gut microbiota, which displayed prebiotic-like activity (Estruel-Amades et al., 2019). Quercetin affected the composition of the gut microbiota in high-fat fed rats, and decreased the ratio of Firmicutes/Bacteroidetes, which indicated that quercetin exerted a mitigating effect on obesity (Etxeberria et al., 2015). In summary, flavonoids can regulate and balance the disordered gut microbiota. Nowadays, various new food, nutraceuticals, and pharmaceuticals have been developed in the market to perform their functional activities. However, little is known about the impacts of most flavonoids on gut microbiota. Based on the antibacterial activity of flavonoids and the fact that hesperetin, naringenin, and quercetin are typical representatives of different subclasses of flavonoids, their effects on gut microbiota are worth exploring. In addition, the differences in the effects of flavonoid aglycones and their corresponding flavonoid monoglucosides and flavonoid diglycosides on the regulation of gut microbiota due to their different structures are also need to be investigated. In this work, the effects of hesperidin, hesperetin-7-O-glucoside, hesperetin, naringin, prunin, naringenin, rutin, isoquercitrin, and quercetin on healthy Chinese volunteers were studied by *in vitro* simulated fermentation technology. The theoretical basis of the differences caused by flavonoids on the structure and metabolism of gut microbiota enables the probable use of flavonoids as a functional food.

**Table 1:** Structural formulae and related information of the nine flavonoids.

Substance	Structural formula	CAS number	Experimental purity
Hesperidin	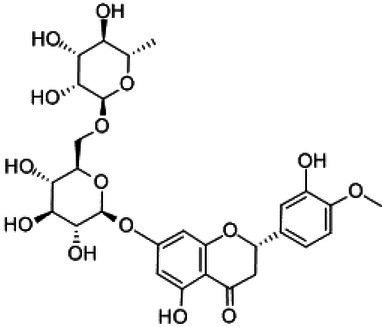	520-26-3	97%
Hesperetin-7-O-glucoside	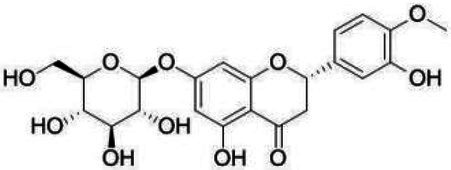	31712-49-9	95.38%
Hesperetin	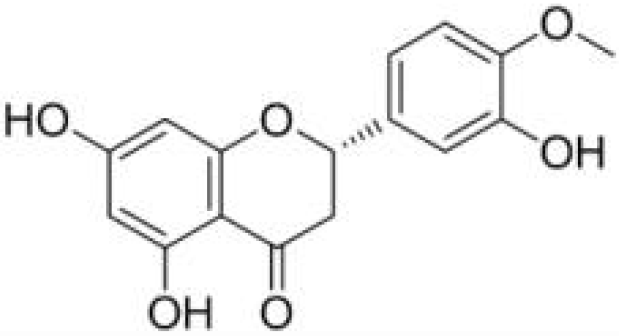	520-33-2	97%
Naringin	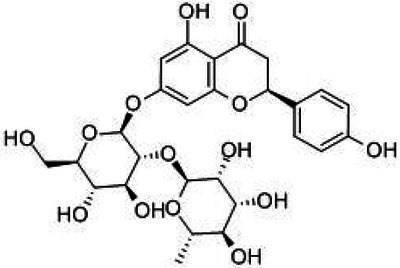	4493-40-7	98%
Prunin	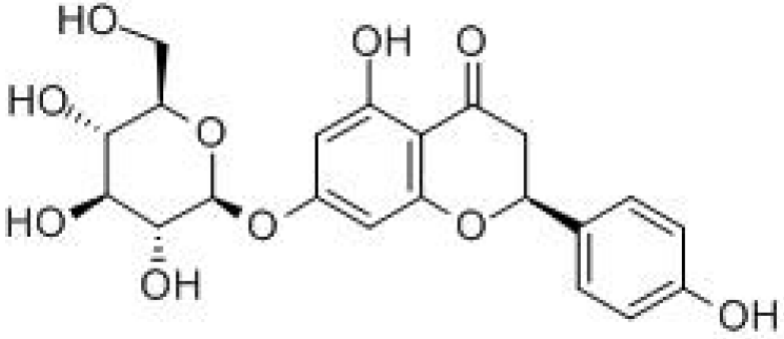	529-55-5	96.78%
Naringenin	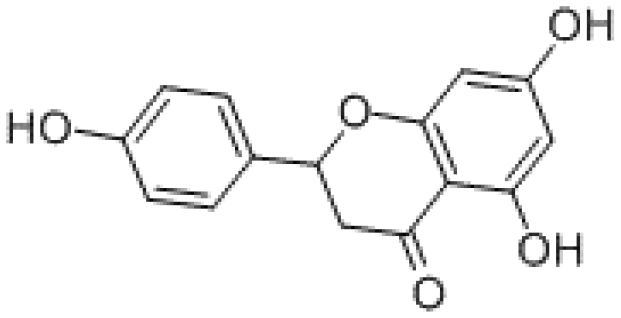	480-41-1	97%
Rutin	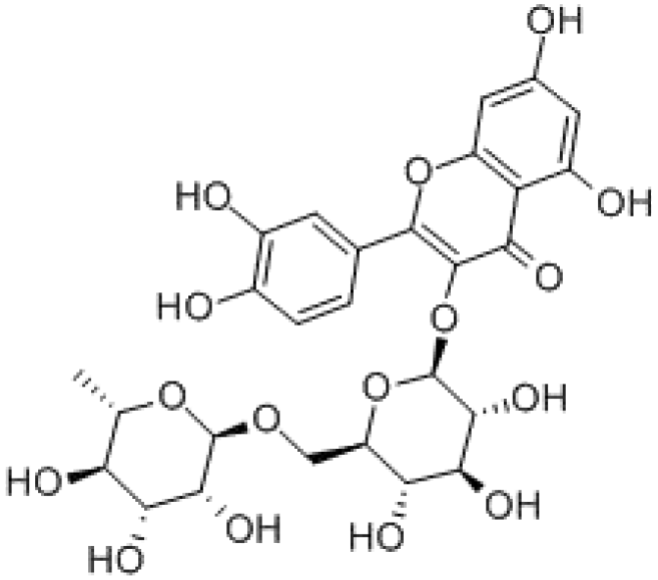	153-18-4	98%
Isoquercitrin	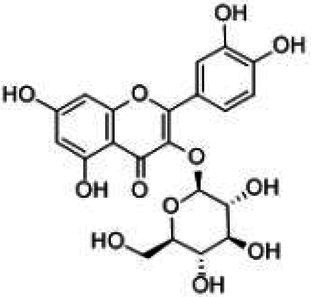	482-35-9	96.51%
Quercetin	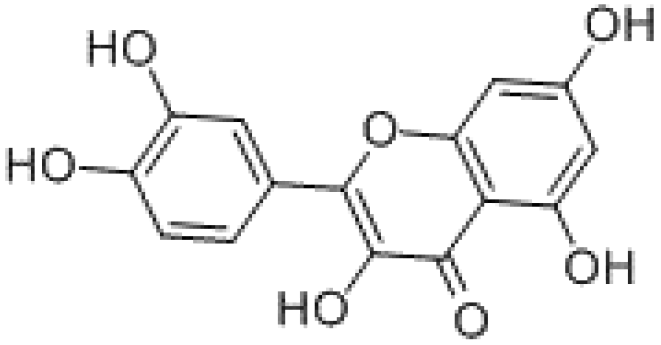	117-39-5	95%

## 2. Materials and methods

### 2.1. Materials and reagents

Hesperetin-7-O-glucoside, prunin, and isoquercitrin were prepared in our laboratory (Ye H. et al., 2022; Hangzhou, China). Hesperidin, hesperetin, naringin, naringenin, rutin, and quercetin were obtained from Aladdin Reagent Co., Ltd. (Shanghai, China). Yeast extract powder, tryptone, crotonic acid, KH_2_PO_4_, K_2_HPO_4_, heme, MgSO_4_, NaCl, CaCl_2_, and L-cysteine were purchased from Sigma (Missouri, United States).

### 2.2. Fecal sample collection

Ten volunteers, five men and five women, aged 20–30 years old (people without no gastrointestinal diseases and not taking antibiotics, prebiotics, probiotics, and other drugs in recent 1 month) were selected from the local healthy population in Hangzhou, Zhejiang Province. All 10 volunteers personally signed the consent form and knew that this was for experimental use. This study was approved by the Ethical Committee of the Hangzhou Center for Disease Control and Prevention (no. 202047). To ensure that the fecal samples collected had little food residue while minimizing contact with oxygen, these volunteers were required to use a sterile fecal sampling box, the stool located in the middle was quickly collected at the time of defecation, and the sample could not be <3 g. Then, the name, age, and date of sampling of the volunteer corresponding to each sample were marked. The fecal samples were kept at 4°C and used in this experiment within 4 h.

### 2.3. Fecal sample pretreatment

Fresh fecal samples of about 0.3 g each were weighed three times from the stool sampling box, and each was placed in a 1.5 mL sterilized centrifuge tubes. Then, these original fecal samples were stored in the refrigerator at −80°C. Thereafter, the fecal samples of 0.8 g were weighed and placed in a 10 mL sterile centrifuge tube and then added with 8 mL of sterile PBS buffer solution. An oscillator was utilized to mix the stool and buffer solution thoroughly, and a sterile filter was used to sieve away large particles. Ultimately, a 10% fecal suspension inoculum was prepared.

### 2.4. Configuration of relevant media

*In vitro* fermentation followed the method of Zhao et al. (2021). The composition of the control medium (Con) was as follows: 10 g tryptone, 2.5 g yeast extract, 2 mL heme solution (5 mg/mL), 1 g L-cysteine, 0.9 g NaCl, 0.45 g K_2_HPO_4_, 0.45 g KH_2_PO_4_, 0.09 g MgSO_4_, and 0.09 g CaCl_2_, which were dissolved in 1 L of deionized water. Immediately after boiling, nitrogen was added to keep the medium level anaerobic. The peristaltic pump dispensed 5 mL into vials, which were sealed up well and sterilized 30 min with high-pressure steam sterilizer at 115°C before use.

Flavonoid medium was prepared by adding hesperidin (S1), hesperetin-7-O-glucoside (S2), hesperetin (S3), naringin (S4), prunin (S5), naringenin (S6), rutin (S7), isoquercitrin (S8), and quercetin (S9) to the control medium. Their final concentration was 4 mg/mL.

### 2.5. *In vitro* fermentation and gas analysis after the fermentation

Each of the nine flavonoids was used as a substrate for *in vitro* fermentation. A blank medium without additional substrate was also added as a control. Fecal samples from 10 donors were added to each of the media mentioned above individually, and three replicate experiments were performed on each media for each donor’s fecal sample. 500 μL of treated 10% fecal suspension inoculum from 10 volunteers was separately inoculated into the above-mentioned medium supplemented with different flavonoids (S1–S9) and control medium (Con) using a disposable sterile syringe in an anaerobic workstation. Each medium was shaken gently, and incubated in an incubator at 37°C for 24 h. The gases produced by *in vitro* anaerobic fermentation of gut microbiota were accumulated in sample vials. According to the method of Ye X. et al. (2022), the total amount of gases and gas composition (CH_4_/NH_3_/H_2_/H_2_S/CO_2_) were measured with a gas analyzer after cooling to room temperature. The injection needle of the gas analyzer delivered the accumulated gases in sample vials to this instrument for analysis, and the highest value of each gas was recorded. And then the gases were re-delivered to vials by the outlet needle, so that the air pressure in sample vials was kept constant. After the gas measurement, the sample vials were opened and the fermentation broth was packed into 1.5 mL centrifuge tubes. Then, each sample was centrifuged at 10,000 rpm for 5 min. The supernatant and precipitate were separated, placed into 1.5 mL centrifuge tubes, and frozen at −80°C for storage.

### 2.6. Determination of short-chain fatty acids

Based on a previous method (Pi et al., 2022; Tian et al., 2022), metaphosphoric acid of 2.5 g was added to ddH_2_O at constant volume to 100 mL, and the mass-to-volume ratio (W/V) of the prepared metaphosphoric acid solution was 2.5%. Then, 0.6464 g of crotonic acid was weighed and added to the metaphosphate solution at constant volume to 100 mL, and the crotonic acid–metaphosphoric acid solution was obtained after even mixing. A total of 100 μL of crotonic acid–metaphosphoric acid solutions was added to 500 μL of fermentation supernatant, followed by fully mixing and acidification for 24 h at −80°C. After acidification, the mixture was centrifuged for 3 min at 10,000 rpm and 4°C. The supernatant was filtered with a 0.22 μm aqueous microporous membrane, and 150 μL of the filtrate was pipetted into the injection vial. It was shaken to expel air bubbles at the bottom of the internal tube for preventing empty aspiration during sample loading.

When the gas chromatograph was ready to sample, the aging process was conducted. The column temperature heating procedure was as follows: the column temperature was 80°C for 1 min, 10°C/min, rising to 190°C, and maintained for 0.50 min. Then, it reached 240°C at the rate of 40°C/min for 5 min. FID detector: 240°C; gasification chamber: 240°C; carrier gas: nitrogen flow rate of 20 mL/min, hydrogen flow rate of 40 mL/min, and air flow rate of 400 ml/min. The editing program started to test the content of different SCFAs.

### 2.7. 16S rRNA gene sequencing of gut microbiota and bioinformatic analysis

Genomic DNA extraction was completed from fecal fermentation broth sediment samples obtained by centrifugation as described above. Genomic DNA was extracted from 10 samples in each experimental group, and the total number of samples tested was 110 when including the control and raw fecal groups. With these extracted genomes verified by electrophoresis as a template, and 341F (5-CCTAYGGGRBGCASCAG-3)/806R (5-GGACTACNNGGGTATCTAAT-3) as upstream and downstream primers, the V3–V4 regions of bacterial 16S rRNA gene were obtained. Purified amplicons were commissioned to Shanghai Meiji Biomedical Technology Co., Ltd. for paired-end sequencing on the Illumina MiSeq PE250 platform. During the use of QIIME2, the optimized sequences were denoised by the sequence noise reduction plugin DADA2 (Callahan et al., 2016; Bolyen et al., 2019). The taxonomic assignment of ASVs was achieved with reference to the resources in the SILVA 16S rRNA gene database (v138). All consensus sequence data of raw fecal and fermentation samples were submitted to the National Center for Biotechnology Information Short Read Archive under accession no. PRJNA874892. The obtained data were further subjected to bioinformatic analysis, and modeling analysis was performed on the microflora and related metabolic data. Bioinformatic analysis was performed on the online platform of Shanghai Meiji Biomedical Technology Co., Ltd.^1^ α-diversity relied on the Ace, Chao, Shannon, and Simpson index assessed at the ASV level. β-diversity was assessed based on Bray-Curtis distance, and expressed using principal coordinate analysis (PCoA). The relative abundance of different groups at the phylum and genus levels was represented by Bar plots. At the genus level, the number of species common and unique to multiple groups was counted using Veen plots. The correlations of the different genera of bacteria contained in the samples with the gases and SCFAs were evaluated using Spearman correlation coefficients, and they were presented in the correlation heatmap.

### 2.8. Statistics and analysis

Results were presented as mean ± SEM (10 independent experiments × 3 parallel experiments). 16S rRNA gene sequencing of gut microbiota included 11 independent experiments. The experimental data were statistically analyzed and plotted by SPSS 26.0 and Origin 2021 software. The Shapiro–Wilk test was used to check whether the data obeyed a normal distribution. For data that obeyed a normal distribution, one-way ANOVA followed by Duncan’s multiple range test was conducted between multiple groups. For data that did not obey a normal distribution, the Kruskal-Wallis test was conducted between multiple groups. *p* < 0.05 indicated statistical significance.

## 3. Results

### 3.1. Effects of flavonoids *in vitro* simulated fermentation on gas production

**Figure 1:**
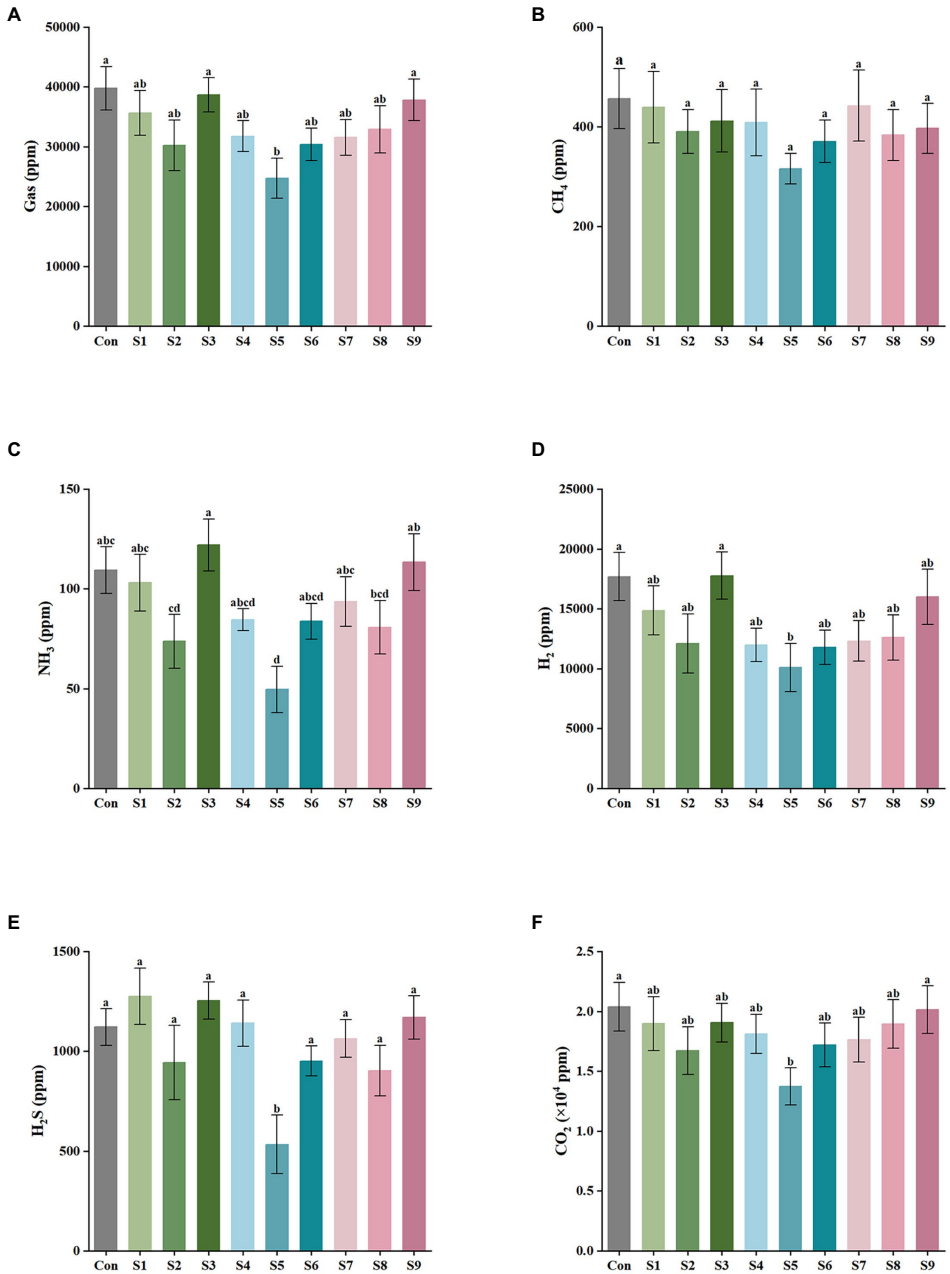
Gas composition and content of external fermentation of fecal microbiota under different substrate conditions. **(A)** The total amount of the five gases, **(B)** CH_4_, **(C)** NH_3_, **(D)** H_2_, **(E)** H_2_S, and **(F)** CO_2._ Data are means ± SEM (10 independent experiments × 3 replication experiments). Different lowercase letters indicate significant differences (*p* < 0.05).

The results of gas production after 24 h of *in vitro* fermentation with the addition of nine flavonoids are shown in Figure 1. The control and experimental media produced a large amount of CO_2_ and H_2_ and a small amount of H_2_S, CH_4_, and NH_3_ after fecal bacteria fermentation for 24 h. As illustrated in Figure 1A, the addition of hesperitin-7-O-glucoside, prunin, and isoquercitrin greatly reduced the total gas production compared with the control, and prunin had a statistically significant effect (*p* < 0.05). Meanwhile, the hesperetin and quercetin groups had no significant difference (*p* > 0.05). As shown in Figures 1B–F, the corresponding decreasing trends of CH_4_, NH_3_, H_2_, H_2_S, and CO_2_ after 24 h of simulated fermentation were also similar to the total, and the decrease in each gas was significant after the addition of pruning (*p* < 0.05).

### 3.2. Effects of flavonoids *in vitro* simulated fermentation on short-chain fatty acid production

**Figure 2:**
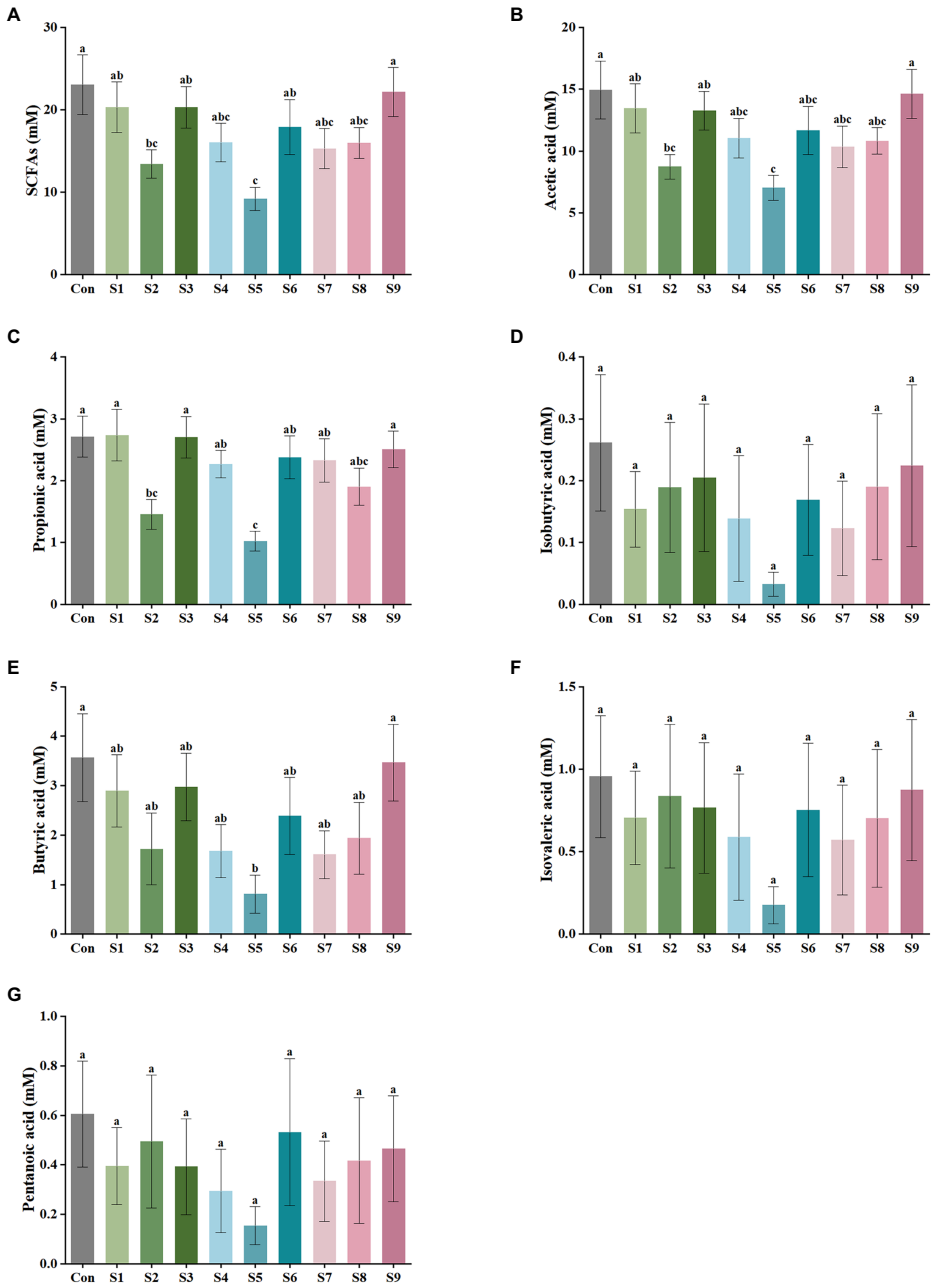
SCFA output of external fermentation of fecal microbiota under different substrate conditions. **(A)** Total SCFA yield, **(B)** Acetic acid, **(C)** Propionic acid, **(D)** Isobutyric acid, **(E)** Butyric acid, **(F)** Isovaleric acid, and **(G)** Pentanoic acid. Data are means ± SEM (10 independent experiments × 3 replication experiments). Different lowercase letters indicate significant differences (*p* < 0.05).

The variations in the content of the six SCFAs in the fermentation liquor in this study were measured, as shown in Figure 2. The highest content of acetic acid was found in the fermentation liquor of fecal samples, followed by those in propionic and butyric acids. The production of isobutyric, isovaleric, and pentanoic acids was the lowest. According to the data of total SCFA production in Figure 2A, the total SCFA content was decreased to 15 mmol/L or even lower by the addition of hesperitin-7-O-glucoside, prunin, rutin, and isoquercitrin compared with the total SCFA content of 23 mmol/L in the control group. The decrease degree of hesperitin-7-O-glucoside and prunin groups was statistically significant (*p* < 0.05). This decreasing trend corresponds to the effect on gas content described above. As displayed in Figures 2B–G, the impact of hesperitin-7-O-glucoside and prunin on the decrease in the content of acetic and propionic acids was significant (*p* < 0.05), and prunin also caused a notable decrease in the level of butyric acid (*p* < 0.05). The effects of the remaining six flavonoids on the production of the three SCFAs were insignificantly different from those of the control group.

### 3.3. Analysis of fecal microbiota composition before and after fermentation

**Figure 3:**
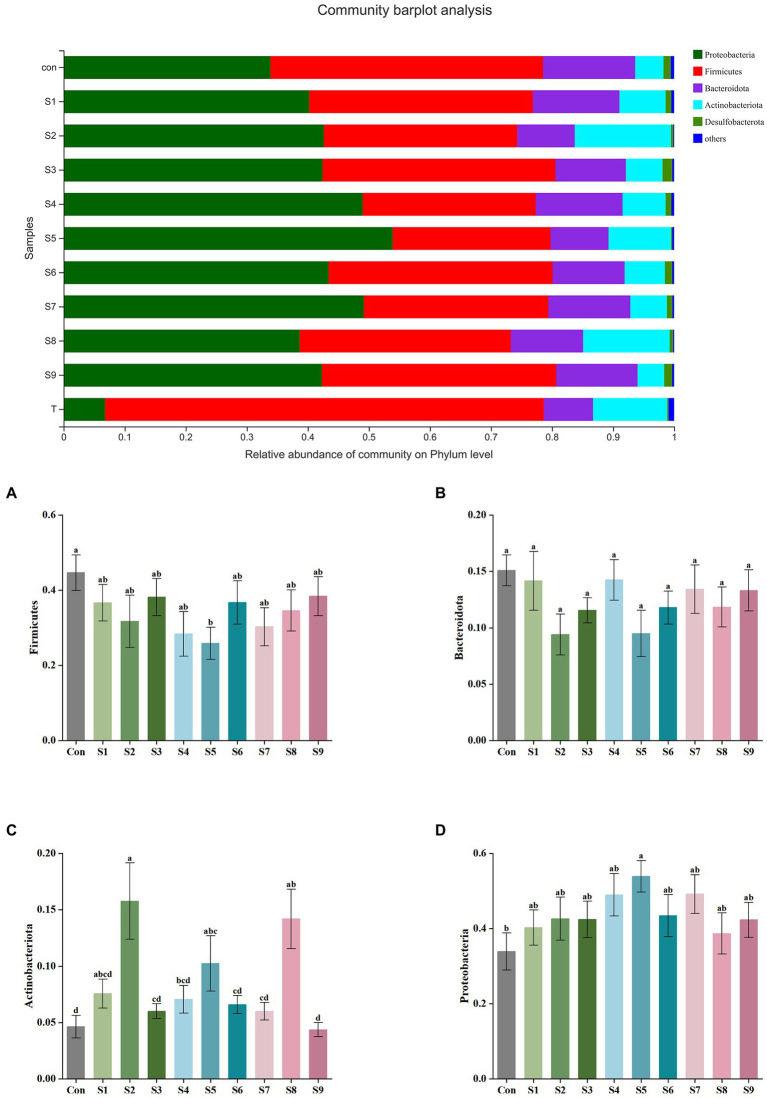
Composition of original fecal microbiota (T) and the abundance of fecal microbiota at the phylum level. Relative abundance of Firmicutes **(A)**, Bacteroidota **(B)**, Actinobacteriota **(C)**, and Proteobacteria **(D)** in fecal microbiota after fermentation. Data are means ± SEM (10 independent experiments). Different lowercase letters indicate significant differences (*p* < 0.05).

**Figure 4:**
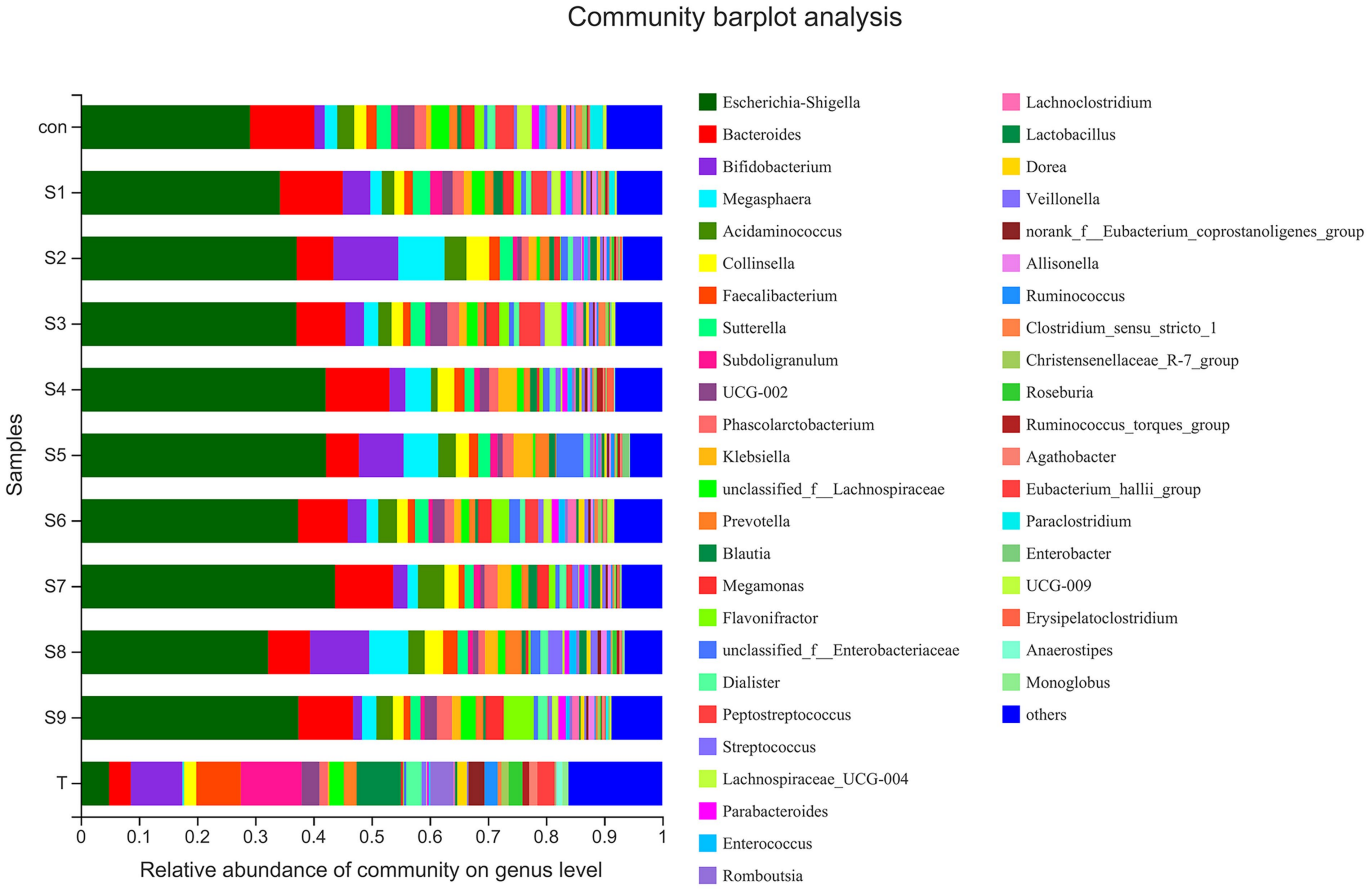
Composition of original fecal microbiota (T) and the abundance of fecal microbiota at the genus level.

**Figure 5:**
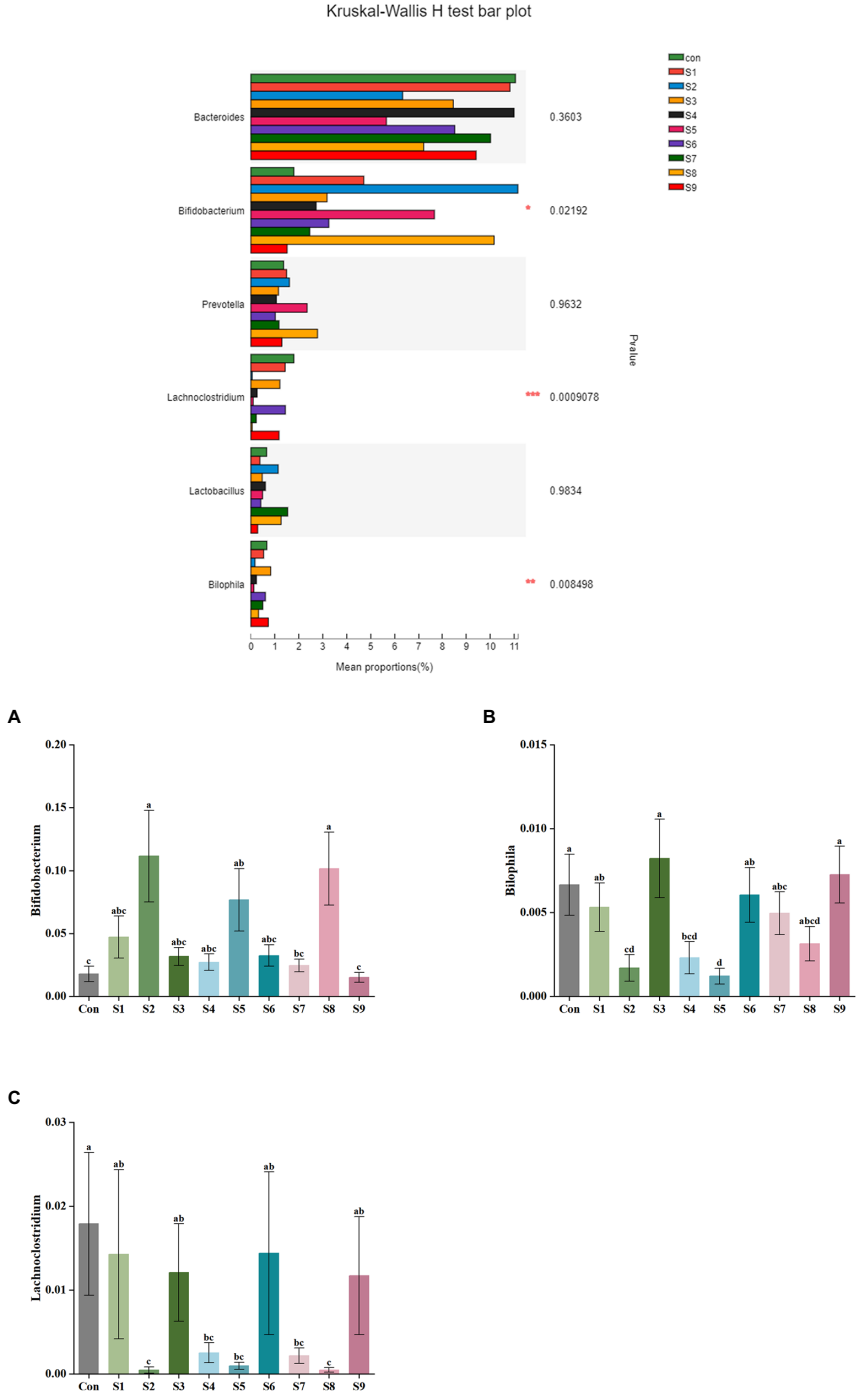
Comparison of fecal microbiota under different substrate conditions at the genus level. Relative abundance of *Bifidobacterium* **(A)**, *Bilophila* **(B)**, and *Lachnoclostridium* **(C)** in fecal microbiota after fermentation. Data are means ± SEM (10 independent experiments). Different lowercase letters indicate significant differences (*p* < 0.05). * 0.01 < *p* ≤ 0.05, ** 0.001 < *p* ≤ 0.01, *** *p* ≤ 0.001.

The changes in fecal microbiota before and after fermentation were determined by 16S rRNA gene sequencing. The abundance of fecal microbiota at the phylum level is shown in Figure 3. The Firmicutes were the most abundant phylum in the raw fecal T-samples, but the abundance of Firmicutes decreased substantially after fermentation, and this abundance in the prunin group was obviously lower (*p* < 0.05). The abundance of Proteobacteria showed an increase in all groups after fermentation, and the abundance of Proteobacteria increased significantly in the prunin group compared to the control group (*p* < 0.05). After fermentation, the abundance of Actinobacteriota in each group except the quercetin group was higher than that in the control group, especially in the hesperetin-7-O-glucoside, prunin, and isoquercitrin groups (*p* < 0.05). The results in Figure 4 show that, compared with after *in vitro* fermentation, *Subdoligranulum* was the most abundant genus in the raw fecal T-samples, followed by *Bifidobacterium*, *Faccalibacterium*, and *Blautia*. At the genus level, we performed analysis focusing on microorganisms with an abundance of more than 1%. After 24 h of fermentation, the abundance of *Bifidobacterium* in the control group was lower than that of raw fecal. Compared with the control group, Figure 5 also shows that the abundance of the probiotics *Bifidobacterium* was improved after the addition of the abovementioned flavonoids, except the quercetin group. Among them, the improvement effect of hesperetin-7-O-glucoside, prunin, and isoquercitrin groups was very significant (*p* < 0.05). Meanwhile, hesperetin-7-O-glucoside, rutin, and isoquercitrin groups all increased the abundance of *Lactobacillus.* Prunin and isoquercitrin groups also enhanced the abundance of *Prevotella* to a greater extent. By contrast, the addition of all nine flavonoids decreased the abundance of *Lachnoclostridium*, and its abundance was significantly low in the hesperetin-7-O-glucoside, naringin, prunin, rutin, and isoquercitrin compared with that in the control group. Adding hesperetin-7-O-glucoside, naringin, and prunin also reduced the relative abundance of *Bilophila*. In addition, adding hesperetin-7-O-glucoside, prunin, and isoquercitrin caused the relative abundance of *Bacteroides* to reduce.

**Figure 6:**
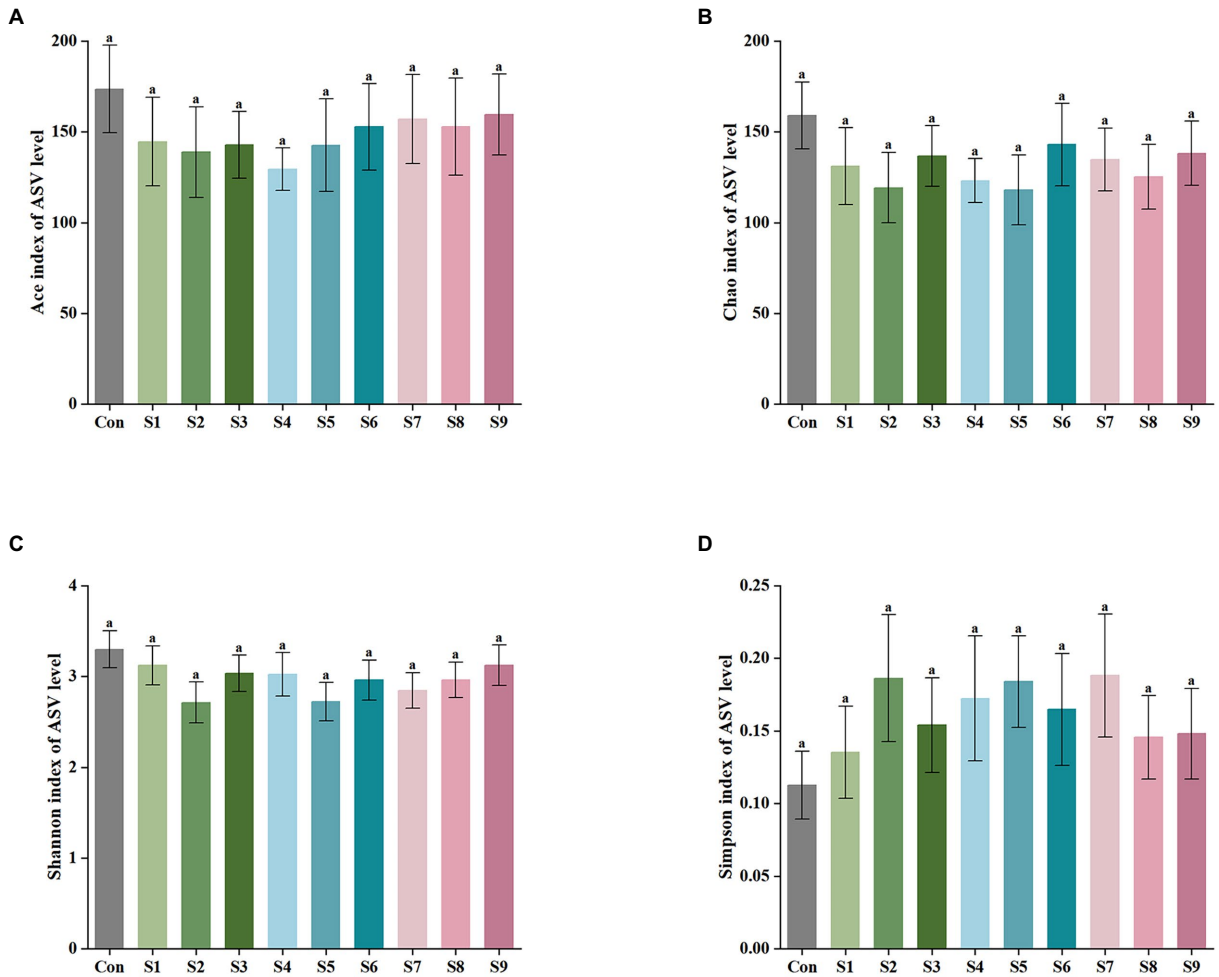
α-Diversity analysis of fecal microbiota after fermentation. **(A)** Ace index of ASV level, **(B)** Chao index, **(C)** Shannon index, and **(D)** Simpson index. Data are means ± SEM (10 independent experiments). Different lowercase letters indicate significant differences (*p* < 0.05).

**Figure 7:**
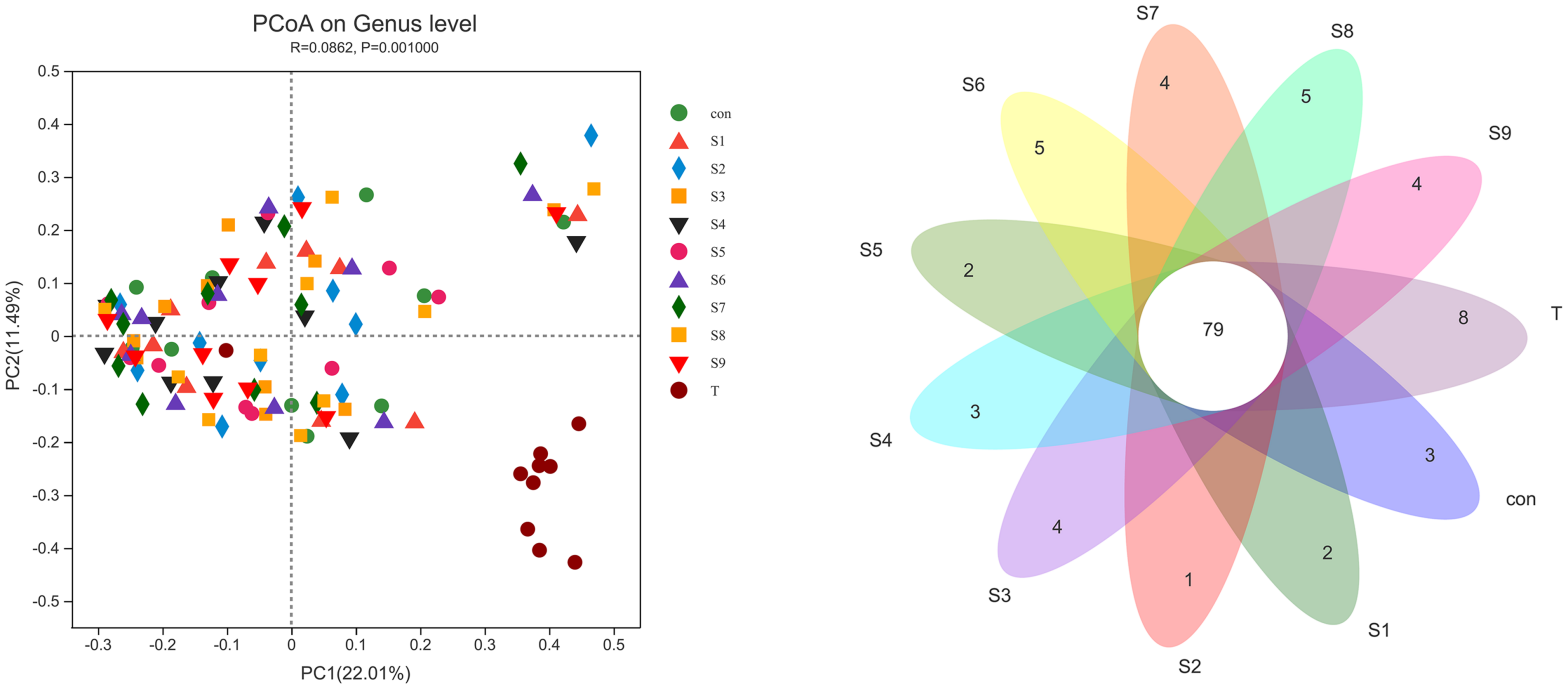
β-Diversity PCoA analysis and Venn diagram analysis of microbiota differences before and after fermentation. *p* < 0.05 indicates a significant difference between groups.

The α-diversity of fecal microbiota was analyzed, and the results are shown in Figure 6. After fermentation with the addition of flavonoids, the Ace index, Chao index, and Shannon index of each group were lower than those of the control group, but all were insignificant (*p* > 0.05). On the contrary, the Simpson index was increased slightly, but the increase was also insignificant (*p* > 0.05). The β-diversity of the gut microbiota and the species Venn diagram were analyzed at the genus level to compare the overall differences in fecal microbiota composition among groups after adding different substrates. As shown in Figure 7, a significant difference existed between the fecal microbiota before and after fermentation (*p* = 0.001). Compared with the group without additional substrate, a certain difference in fecal microbiota with flavonoids was observed after fermentation, but it was insignificant. A total of 79 species of the same bacteria before and after fermentation were found, and the microbiota after fermentation and the raw fecal microbiota differed in eight species.

### 3.4. Correlation analysis between fecal microbiota and metabolites

**Figure 8:**
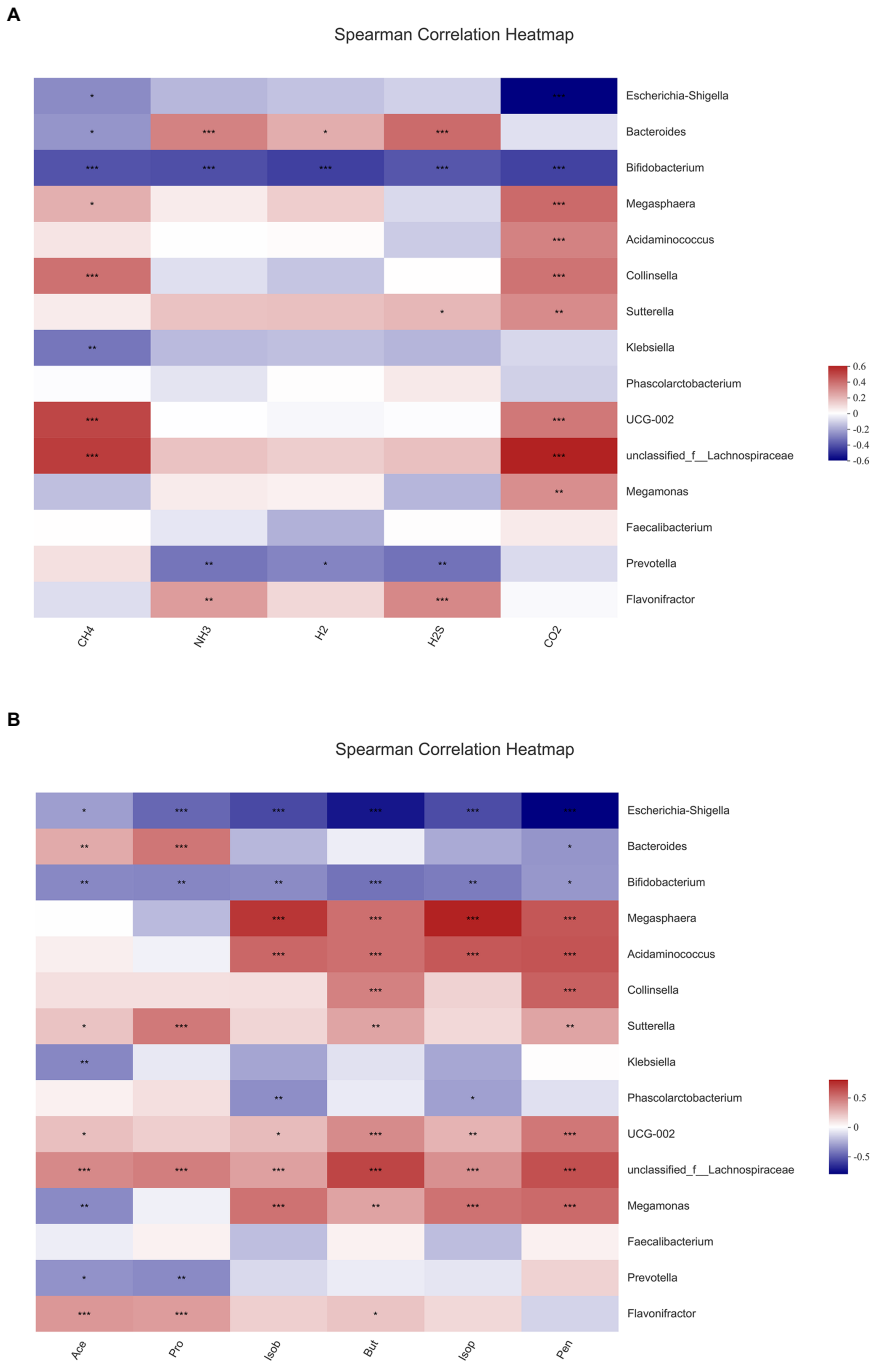
Correlation analysis between gut microbiota and gas **(A)**, short-chain fatty acids and **(B)** in fermentation samples. Data are means ± SEM (10 independent experiments). * 0.01 < *p* ≤ 0.05, ** 0.001 < *p* ≤ 0.01, *** *p* ≤ 0.001.

In this experiment, the relationship of gut microbiota with gas and SCFAs was analyzed with heatmaps and Spearman correlation coefficients, and the results are displayed in Figure 8. The bacteria with the top 15 abundance at genus level were screened. All gases and SCFAs were collectively influenced by a large number of bacteria, and the bacteria significantly affected CO_2_ production, followed by CH_4_. In terms of individual bacteria, *Bifidobacterium* was the genus of bacteria that significantly affected production of all gases and SCFAs, and all were negatively correlated. *Prevotella* was strikingly negatively correlated with NH_3_, H_2_, H_2_S, acetic acid, and propionic acid. *Flavonifractor* then exhibited a significant positive correlation with NH_3_, H_2_S, acetic acid, propionic acid, and butyric acid. A remarkable positive correlation was observed between *Bacteroidetes* and NH_3_, H_2_, H_2_S, acetic acid, and propionic acid, and a negative correlation was found with CH_4_ and pentanoic acid. *Escherichia-Shigella* had the highest abundance, and it was significantly negatively correlated with CH_4_, CO_2_, and all six SCFAs. However, *Faccalibacterium* had no significant correlation with any gases or SCFAs.

## 4. Discussion

The type and content of intestinal gases, as an important metabolite of intestinal bacteria, play a key part in the host, which are principally affected by flora and diet (Kalantar-Zadeh et al., 2019). Excessive amounts of gas produced in the intestine can cause discomfort such as bowel sounds and abdominal distension (Chang et al., 2020). Thus, in this experiment, the similarities and differences in the metabolic gas production of different flavonoids such as hesperidin (S1), hesperetin-7-O-glucoside (S2), hesperetin (S3), naringin (S4), prunin (S5), naringenin (S6), rutin (S7), isoquercitrin (S8), and quercetin (S9) at a same final concentration (4 mg/mL) were investigated by *in vitro* simulated fermentation technology. As observed, all the nine flavonoids above could reduce the total gas production, and the reduction effect of hesperitin-7-O-glucoside, prunin, and isoquercitrin was obvious; especially, the prunin group statistically reduced the total gas production (*p* < 0.05), and the reduction effect of CO_2_, NH_3_, H_2_S, and CH_4_ (*p* < 0.05) were also all significant (Figure 1). Relevant studies have shown that NH_3_ and H_2_S produced by fermentation of gut microbiota is related to gastrointestinal disorders, including Crohn’s disease and ulcerative colitis (Singh and Lin, 2015). Furthermore, a close correlation existed between the decrease in gas content and the changes in gut microbiota. The results demonstrate that the nine flavonoids above (particularly hesperitin-7-O-glucoside, prunin, and isoquercitrin) could regulate the gut microbiota, which could further adjust intestinal gas composition. In addition, the modulating effect of prunin should not be underestimated.

SCFAs are another vital metabolite of intestinal bacteria, and they are derived from carbohydrates that the body cannot digest on its own. The effect of flavonoids on SCFA production is mainly due to the direct effect of flavonoids on the gut microbiota which produces SCFAs. For example, baicalin treatment could increase the abundance of *Bifidobacterium* and *Ruminococcaceae* in the intestine of hypertensive rats, and both bacteria could produce different SCFAs (Wu et al., 2019). These SCFAs not only can be directly used by the body to play important physiological regulatory roles, such as influencing energy balance and glucose homeostasis directly through the central pathway or by the gut-brain axis, but also can be used as energy sources (Luo et al., 2022). Current studies have shown that SCFAs are critical for the metabolism of host and the activity of gut microbiota (Serino, 2019). Thus, in this research, the content of six common SCFAs was quantified, and the results of the assay focused on the three SCFAs with high contents above (Figure 2). The decrease in the content of SCFAs was also inextricably correlated with the changes in the gut microbiota structure. The addition of hesperetin-7-O-glucoside, prunin, and isoquercitrin resulted in a greater decrease in the total SCFA production. This finding implies that the number and abundance of intestinal flora had been regulated by these substrates, and the flavonoids hesperetin-7-O-glucoside, prunin, and isoquercitrin had various physiological activities such as bactericidal, antiviral, and anti-inflammatory. The production of acetic, propionic, and butyric acids was significantly decreased by the addition of prunin (*p* < 0.05). These results suggest that prunin had excellent antibacterial and anti-inflammatory effects. Many studies have proven that flavonoids have broad-spectrum antibacterial effects; for example, high-flavonoid apple would inhibit the growth of *Lactobacillus* (Espley et al., 2014), and ellagitannins had inhibitory effects on *Bacillus cereus* and *Candida albicans* (Nohynek et al., 2006). *Smilax china* L. flavonoid could reduce SCFAs in obese mice (Li et al., 2021), and this result is consistent with the fact that nine flavonoids reduced the total SCFAs to different degrees in this study. However, in many studies, flavonoids have been reported to increase the concentration of SCFAs due to the increase in the abundance of beneficial bacteria (Wu et al., 2019; Xuan et al., 2020). In contrast, the decrease in the content of SCFAs in the present study is attributed to the broad-spectrum inhibition of flavonoids. That means, flavonoids have certain inhibitory effects on all intestinal bacteria, including causing a decrease in the abundance of SCFA-producing bacteria (Espley et al., 2014; Gwiazdowska et al., 2015). This reason ultimately led to an overall decrease in the content of SCFAs.

Gut microbiota ferments human dietary intake of various nutrients to promote nutrient absorption and significantly influence the biotransformation and metabolic processes of the ingested active substances; thus, it regulates human health (Cotillard et al., 2013). In the digestive tract from stomach to colon, the colon contains the highest number and activity of gut microbiota (Rooks and Garrett, 2016). Meanwhile, flavonoids have a long residence time in the colon and can interact with a wide variety of intestinal flora therein; thus, they modulate the structure of the flora (Braune and Blaut, 2016). The regulatory action of flavonoids on the gut microbiota can be divided into two main aspects. On the one hand, flavonoids can be metabolized by the gut microbiota, and the flavonoids act as substrates in a series of catalytic reactions with various enzyme systems produced by the gut microbiota; as a result, the bacteria involved in these reactions will exhibit a tendency to grow (Lu et al., 2018). On the other hand, flavonoids can affect the cell membranes of some bacteria such as *Escherichia coli* and *Staphylococcus aureus*; for example, they directly disrupt the lipid bilayer of the cell membrane or alter the cell membrane permeability; ultimately, the reproduction of these bacteria is inhibited (Xie et al., 2015). We analyzed the differences in the taxonomic profiles of fecal microbiota before and after fermentation to clarify the modulation of gut microbiota structure by the nine flavonoids above. At the phylum level, this phenomenon was mainly reflected by the addition of the nine flavonoids that caused, to varying degrees, a reduction in the abundance of Firmicutes and an enhancement in the abundance of Actinobacteriota (Figure 3). This finding is consistent with that of previous studies in which the combined intervention of quercetin and resveratrol in rats markedly lowered the abundance of Firmicutes (Zhao et al., 2017). Among the bacteria involved in the metabolic transformation of flavonoids, Actinobacteriota accounted for a large proportion (Braune and Blaut, 2016). The remarkable rise in the abundance of Actinobacteriota in the hesperetin-7-O-glucoside and isoquercitrin groups may indicate that the two substrates have been more fully metabolized. Also, the addition of flavonoids increased the abundance of Proteobacteria. This phenomenon suggests that flavonoids promote the growth of Proteobacteria, which contains most of the harmful bacteria (Shin et al., 2015). This result supports the previous finding that supplementation with mulberry leaf flavonoids promoted an increase in the abundance of Proteobacteria in the gut microbiota of calves, and the specific promotion mechanism needs to be further investigated (Bi et al., 2017). However, the final conclusion of this study showed that mulberry leaf flavonoids could improve the gut health of calves. The main reason is that the researchers took into account the changes induced by mulberry leaf flavonoids at the genus level. Therefore, we also need further analysis at the genus level to determine more comprehensively whether the addition of flavonoids is beneficial to the gut microbiota.

An increasing number of studies have shown that diversity of intestinal flora tends to be positively associated with human health and high levels of beneficial bacteria are more conducive to health. Consequently, in this study, the analysis of fecal microbiota composition at the genus level focused on the abundance of beneficial bacteria. The abundance of *Bifidobacterium*, *Lactobacillus*, and *Prevotella* was relatively enhanced after fermentation with the addition of hesperetin-7-O-glucoside, prunin, and isoquercitrin (Figure 5). *Bifidobacterium*, *Lactobacillus*, and *Prevotella* are all probiotics in human intestinal tract, and they have the functions of promoting absorption and digestion in the gastrointestinal tract, biological barrier function, and regulation of immunity (Odamaki et al., 2016; Milani et al., 2017). Currently, many studies have demonstrated that flavonoids can contribute to the increase in the abundance of these beneficial bacteria. The reason is mainly the involvement of these beneficial bacteria in the metabolism of flavonoids, particularly flavonoid monoglucosides. Icariside I is also a flavonoid monoglucoside. Oral administration of icariside I to tumor-bearing mice has been reported to significantly enhance the abundance of *Lactobacillus* and *Bifidobacterium* in the cecal contents (Chen et al., 2021). *Bifidobacterium* and *Lactobacillus* have been reported to prove β-glycosidase activity, so they can perform hydrolysis reaction on flavonoid monoglucosides (Ávila et al., 2009). These studies fully explain the substantial increase in the abundance of *Bifidobacterium* and *Lactobacillus* in the three flavonoid monoglucoside groups, hesperetin-7-O-glucoside, prunin, and isoquercitrin. In addition to beneficial bacterial changes, we noted the effect of the nine flavonoids on some harmful bacteria. *Bilophila*, which can produce lipopolysaccharide, not only fails to promote host health but also aggravates inflammation and eventually leads to metabolic disorders (Lu et al., 2021). The results show that hesperetin-7-O-glucoside, naringin, and prunin significantly decreased the abundance of *Bilophila*, which indicates that the three flavonoids might possess better effects on anti-inflammation. Moreover, the addition of flavonoids resulted in a relative reduced abundance of *Bacteroides* and *Lachnoclostridium*. Certain strains of *Bacteroides* have been shown that they can produce harmful metabolites, such as *Bacteroides fragilis* and *Bacteroides thetaiotaomicron* (Onoue et al., 1997). The present study also supported previous related studies *in vivo* and *in vitro* that flavonoids inhibited the growth of *Bacteroides* (Zhang et al., 2013; Wang et al., 2020). Due to their ability to regulate the gut-liver axis, flavonoids have an antihyperlipidemic effect. For example, hyperlipemia in mice was alleviated by the intake of flavonoids, while *Lachnoclostridium* was also significantly reduced (Duan et al., 2021). The significant decrease in *Lachnoclostridium* abundance in the current study is also consistent with this result. In conclusion, on the one hand, the nine flavonoid compounds above, especially hesperetin-7-O-glucoside, prunin, and isoquercitrin, can induce beneficial intestinal bacteria to become abundant while decreasing the percentage of harmful intestinal bacteria. On the other hand, they possessed the function of improving the imbalance of intestinal flora and could reduce the influence of other factors on intestinal flora. It needed to be particularly emphasized that the effects of hesperetin-7-O-glucoside, prunin, and isoquercitrin on the gut microbiota were more pronounced than those of the remaining six flavonoids. It is still mainly because these three substrates belong to flavonoid monoglucosides, which are structurally characterized by containing only a single glucose group. In the studies by Makino et al., direct oral administration of quercetin and its various O-glycoside derivatives to rats showed that the bioavailability of isoquercitrin was higher than that of quercetin, whereas the bioavailability of rutin was lower than that of quercetin (Makino et al., 2009, 2013). Combined with the findings that flavonoid monoglucosides, such as puerarin-7-O-glucoside and calycosin-7-O-β-D-Glucoside, had a longer residence time in the blood plasma than their corresponding flavonoid aglycones (Jiang et al., 2008; Ruan et al., 2015), we presumed that the bioavailability of flavonoid monoglucosides might be generally higher. Many studies have confirmed that one of the factors affecting the bioavailability of flavonoids is the interaction between flavonoids and gut microbiota (Hanske et al., 2009; Zhang et al., 2021; Baky et al., 2022). We thus suggest that the single glucose group contained in the structures of hesperetin-7-O-glucoside, prunin, and isoquercitrin make their interaction with gut microbiota more pronounced than the corresponding flavonoid aglycones and flavonoid diglycosides. However, neither α-diversity nor β-diversity of the fecal microbiota composition showed significant differences in each group after fermentation. Therefore, the addition of the nine flavonoids did not result in significant differences in community distribution among the groups, and the richness and diversity of species in the community did not change significantly. In several previous *in vivo* studies, flavonoids were found to increase the α-diversity and β-diversity of gut microbiota in mice (Peng et al., 2020; Xuan et al., 2020). However, the results of this study showed no significant effect of the nine flavonoids on the diversity of gut microbiota. We speculate that the main reason for this is the effect of the broad-spectrum inhibition of flavonoids during the *in vitro* study, and their antibacterial effects depend on the concentration of flavonoids.

Different fecal bacteria produce different metabolites when fermenting different substrates, and the similarities and differences of metabolites can affect the differences in bacterial abundance. The fermentation substrate affects the composition of bacterial communities after fecal bacteria fermentation, which leads to the similarities and differences of metabolites such as gases and SCFAs. We made a correlation analysis between fecal flora and metabolites to account for the changes in the two major metabolite groups of intestinal flora. All six SCFAs were obviously negatively associated with *Bifidobacterium*. Acetic and propionic acids also showed a significant negative relationship with *Prevotella* and a significant positive relationship with *Bacteroides* and *Flavonifractor*. After fermentation with the addition of nine flavonoids, the abundance of *Bifidobacterium* and *Prevotella* were relatively rose, while the abundance of *Bacteroides* and *Flavonifractor* were reduced, especially in the hesperetin-7-O-glucoside, prunin, and isoquercitrin groups. Thus, the concentration of SCFAs was decreased in all the groups with the addition of nine flavonoids, and the decrease was higher in the three groups. All gases were also significantly and negatively correlated with *Bifidobacterium*. Meanwhile, NH_3_, H_2_, and H_2_S were significantly negatively correlated with *Prevotella* and with *Bacteroides*. This finding correlates with the decrease in gas content after the addition of flavonoids. In summary, the community structure analysis also explains the decrease in gas and SCFA content after fermentation with the addition of the nine flavonoids mentioned above.

## 5. Conclusion

In this study, we investigated the similarities and differences in metabolic gas and SCFA production of different flavonoids (hesperidin, hesperetin-7-O-glucoside, hesperetin, naringin, prunin, naringenin, rutin, isoquercitrin, and quercetin) at 4 mg/mL in the fecal microbiota of 10 healthy Chinese individuals using an *in vitro* simulated fermentation technology. The results reveal that hesperetin-7-O-glucoside, prunin, and isoquercitrin can noticeably increase the relative abundance of intestinal probiotics, among which the increase in *Bifidobacterium* is the most significant, and they can decrease the relative abundance of *Lachnoclostridium* and *Bilophila*. A certain amount of SCFAs and metabolic gases are produced, and these metabolites are intimately connected with the gut microbiota composition. This work provides a theoretical reference for the use of flavonoids to be a functional food.

## Data availability statement

The datasets presented in this study can be found in online repositories. The names of the repository/repositories and accession number(s) can be found in the article/supplementary material.

## Ethics statement

The studies involving human participants were reviewed and approved by the Ethics Committee of the Hangzhou Center for Disease Control and Prevention (no. 202047). The patients/participants provided their written informed consent to participate in this study. Written informed consent was obtained from the individual(s) for the publication of any potentially identifiable images or data included in this article.

## Author contributions

LP: data curation, formal analysis, investigation, and writing-original draft. HY: data curation, formal analysis, validation, and visualization. XP: methodology and conceptualization. WL: software and resources. ZW: supervision. YZ: funding acquisition. JZ: methodology, project administration, and review. All authors contributed to the article and approved the submitted version.

## Funding

This work was supported by the National Natural Science Foundation of China (grant no. 31600639) and Key Research and Development Program of Zhejiang Province (2019C01082).

## Conflict of interest

The authors declare that the research was conducted in the absence of any commercial or financial relationships that could be construed as a potential conflict of interest.

## Publisher’s note

All claims expressed in this article are solely those of the authors and do not necessarily represent those of their affiliated organizations, or those of the publisher, the editors and the reviewers. Any product that may be evaluated in this article, or claim that may be made by its manufacturer, is not guaranteed or endorsed by the publisher.

## Footnotes

^1^ www.Majorbio.com
